# Self-supervised classification of subcellular morphometric phenotypes reveals extracellular matrix-specific morphological responses

**DOI:** 10.1038/s41598-022-19472-2

**Published:** 2022-09-12

**Authors:** Kin Sun Wong, Xueying Zhong, Christine Siok Lan Low, Pakorn Kanchanawong

**Affiliations:** 1 Department of Biomedical Engineering, National University of Singapore, Singapore, 117411 Republic of Singapore; 2 Mechanobiology Institute, National University of Singapore, Singapore, 117411 Republic of Singapore

**Keywords:** Computational biology and bioinformatics, Classification and taxonomy, Data processing, Image processing, Machine learning

## Abstract

Cell morphology is profoundly influenced by cellular interactions with microenvironmental factors such as the extracellular matrix (ECM). Upon adhesion to specific ECM, various cell types are known to exhibit different but distinctive morphologies, suggesting that ECM-dependent cell morphological responses may harbour rich information on cellular signalling states. However, the inherent morphological complexity of cellular and subcellular structures has posed an ongoing challenge for automated quantitative analysis. Since multi-channel fluorescence microscopy provides robust molecular specificity important for the biological interpretations of observed cellular architecture, here we develop a deep learning-based analysis pipeline for the classification of cell morphometric phenotypes from multi-channel fluorescence micrographs, termed SE-RNN (residual neural network with squeeze-and-excite blocks). We demonstrate SERNN-based classification of distinct morphological signatures observed when fibroblasts or epithelial cells are presented with different ECM. Our results underscore how cell shapes are non-random and established the framework for classifying cell shapes into distinct morphological signature in a cell-type and ECM-specific manner.

## Introduction

Cell morphology depends on the interplay between cell autonomous properties and the spatiotemporal properties of the extracellular environment^1^. The interaction between cells and the extracellular matrix (ECM) has long been known to exert strong influence on cell morphology^2^. Various ECM types such as collagens, fibronectin, vitronectin and laminin are differentially recognized by specific integrin αβ-receptors^3,4^. Since different cell types express variable combination of integrins and possess different configurations of downstream signalling pathways, distinctive cell-type specific morphologies and migration characteristics are typically observed when cells are presented with purified ECM^2^. Additionally, beyond cell-ECM adhesion, cell morphology has also been shown to play major roles in numerous other cellular activities, suggesting that cell morphology could effectively encode key information on the underlying biochemical signalling states of the cells^1^. For example, dynamic cell morphology has been observed during epithelial-to-mesenchymal transition (EMT), a biological process whereby polarized epithelial cells undergo multiple biochemical alterations to assume a mesenchymal cell phenotype, which includes the disruption of cell–cell interactions, a more variable cell shape, and motile characteristics as single cells^5^. Cell morphology has thus been broadly utilized as a key read-out in high-throughput imaged-based assay for uncovering biological principles^1^ or therapeutic applications^2,6,7^.

Distinct morphological patterns observed for different cell types under various conditions such as different ECM are often readily perceived by human experts at qualitative level^8^. While this may be indicative of latent causal linkages between ECM-dependent signalling and cell morphology, it has been challenging to quantitatively analyse such morphological features. Additionally, cells are compositionally complex, and multi-channel or multi-modality data (e.g. fluorescence or phase-contrast microscopy) are regularly utilized in biological research. Thus, there is a need for an analysis workflow capable of addressing the complex multi-dimensional morphology of cell shapes. Although cell morphology has been traditionally quantified using geometric parameters^9,10^ that describe the size and shape of the cells and salient organelles such as the nuclei, their limitations inherent in its relative simplicity has motivated continual development of machine learning (ML)-based approaches for cell morphology quantification^11^. However, traditional ML-based approaches require a laborious step of manual feature engineering, and thus may not scale well given the high data throughput rate of contemporary microscopy techniques^12^. Recently, deep learning (DL) has emerged as a highly successful approach for image analysis tasks, offering superior performances in multiple image-based analysis^13–16^. In DL, raw data is transformed into successive layers of representations to ‘learn’ complex functions using multiple processing layers^17^. Unlike traditional ML, DL-based models acquire features from data via a learning procedure and does not require human intervention in the extraction of features, also known as feature engineering. Given the nature of biomedical image datasets where novel features cannot always be anticipated, a DL-based analysis workflow that can learn high quality features without the use of manual annotations should be highly beneficial.

Notably, numerous previous studies had made use of cluster analysis to study biological phenotypes such as cell density-based phenotype^18^ or cellular mode-of-action^19^, distinguishing between disaggregated cells^20^, and cell shape modes^21^. While distinct morphodynamics of cells on different ECMs have been well documented^22–25^, a reference-free, image-based quantitative analysis of how cell morphology responds to ECM has been limited because of the lack of information on which subcellular features and components selectively align with the specific ECM. In this study, we therefore sought to develop an integrated analysis workflow capable of classifying multi-channel fluorescence micrograph images of cells, using the morphological variety of different cell types on different ECM as a test case. We developed a residual neural network with squeeze-and-excite blocks (SE-RNN) and demonstrate its ability to classify multi-channel fluorescence images of epithelial and fibroblast cells on multiple ECMs.

## Results

### Distinct ECM-dependent cell-type specific morphological features

**Figure 1:**
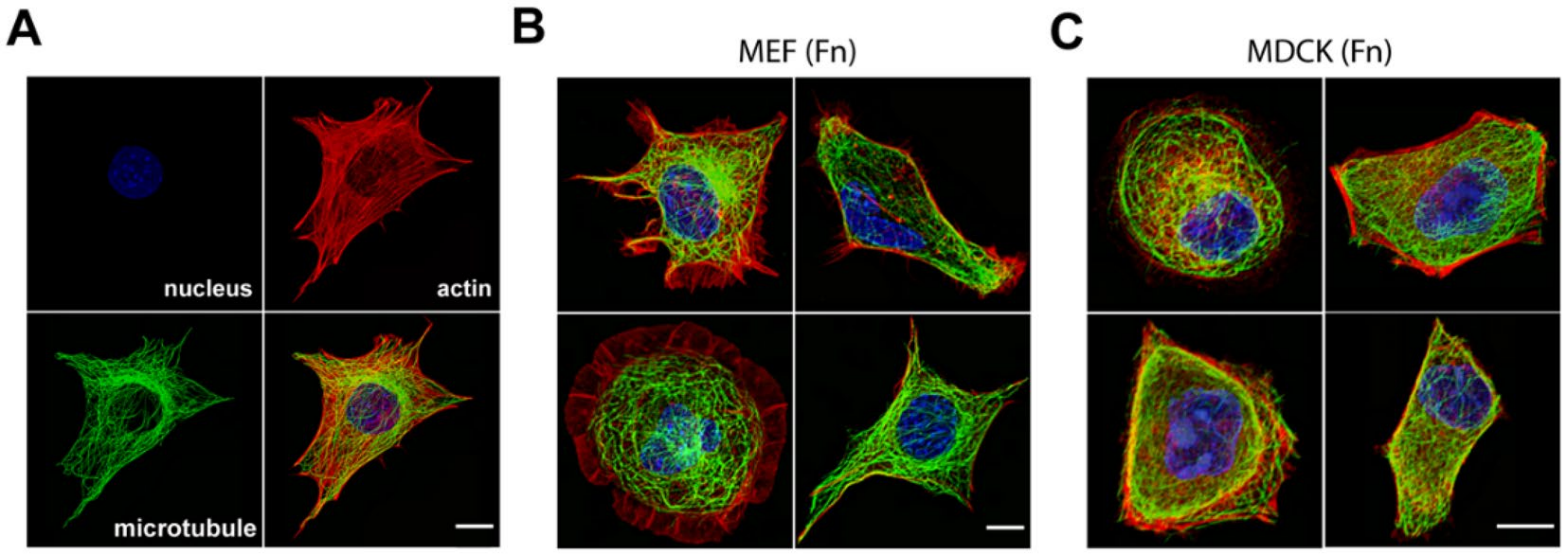
Diverse subcellular morphology in fibroblast and epithelial cells. (**A**) Representative images of subcellular structures used for classification in this study as imaged in Mouse Embryonic Fibroblast (MEF) plated on Fibronectin. (**B**,**C**) Multi-channel Immunofluorescence micrograph of different cell morphology observed in (**B**) mesenchymal (MEF) and (**C**) epithelial Madin-Darby Canine Kidney (MDCK) cells. See also Supplementary Fig. 1 for all the 12 classes and the representative image for each class. Channels: Nucleus (blue), microtubule (green), actin filament (red). Scale bar: 5 μm.

To investigate distinct cell-type specific morphologies, we chose the Madin-Darby Canine Kidney (MDCK II) and Mouse Embryonic Fibroblasts (MEF) as representative epithelial and fibroblast cell models, respectively. Fibronectin (FN), vitronectin (VN), collagen I (C1), collagen IV (C4), mouse Laminin (LN), and laminin-10 (LN10) were chosen as representative ECMs as these are known to engage different combination of integrin ECM receptors^26–33^. Since cell shape and cell mechanics are predominantly dependent on the architecture of cytoskeletal and organelle networks^2,34,35^ we focused our analysis on multi-channel fluorescence micrographs of the actin filaments, microtubules, and the nucleus. Laser-scanning confocal microscope images of MDCK and MEF were acquired at diffraction-limited resolution, with DAPI staining for nucleus, Alexa Fluor 488 conjugated antibody for tubulin, and Alexa Fluor 568 phalloidin for F-actin, respectively. As shown in both Fig. 1 and Supplementary Fig. 1, epithelial and fibroblast cells exhibit diverse yet distinctive morphological differences. For example, on multiple ECM, the epithelial MDCK cells tend to develop approximately polygonal morphology with multiple tapered protrusions, typically helmed by actin stress fibres. In contrast, for fibroblast cells, prominent actin-rich protrusions were observed on collagen I and collagen IV. Moreover, on Laminin-10, actin-rich lamellipodia form broad rim of the cells that exclude microtubules.

We next performed a statistical analysis to investigate whether the observed morphological differences are statistically significant (Supplementary Table 1). We used principal component analysis (PCA) to extract the first two principal components for the entire dataset that preserve the global morphological variance of the datasets. We conducted F-test on both the MDCK and MEF datasets for every ECM, with the results indicating a large variance between both sample means with extreme level of confidence. The lowest F-statistic score observed was about 14.2 for laminin-10, which indicates that the variance between the MDCK and MEF morphological distribution is extremely large. The highest p-value was also observed for laminin-10 at an extremely small value of approximately 10^–4^, yielding strong evidence against the null hypothesis (i.e. any observed difference between the two distributions is not due to chance). Our analysis indicates that the responses of these two cell types in the same ECM environment are highly likely to be significantly different. As computational tools capable for classifying such multi-dimensional morphological complexity have not been available to our knowledge, we next sought to develop this capability.

### Morphological classification by residual neural network with squeeze-and-excitation (SE-RNN)

**Figure 2:**
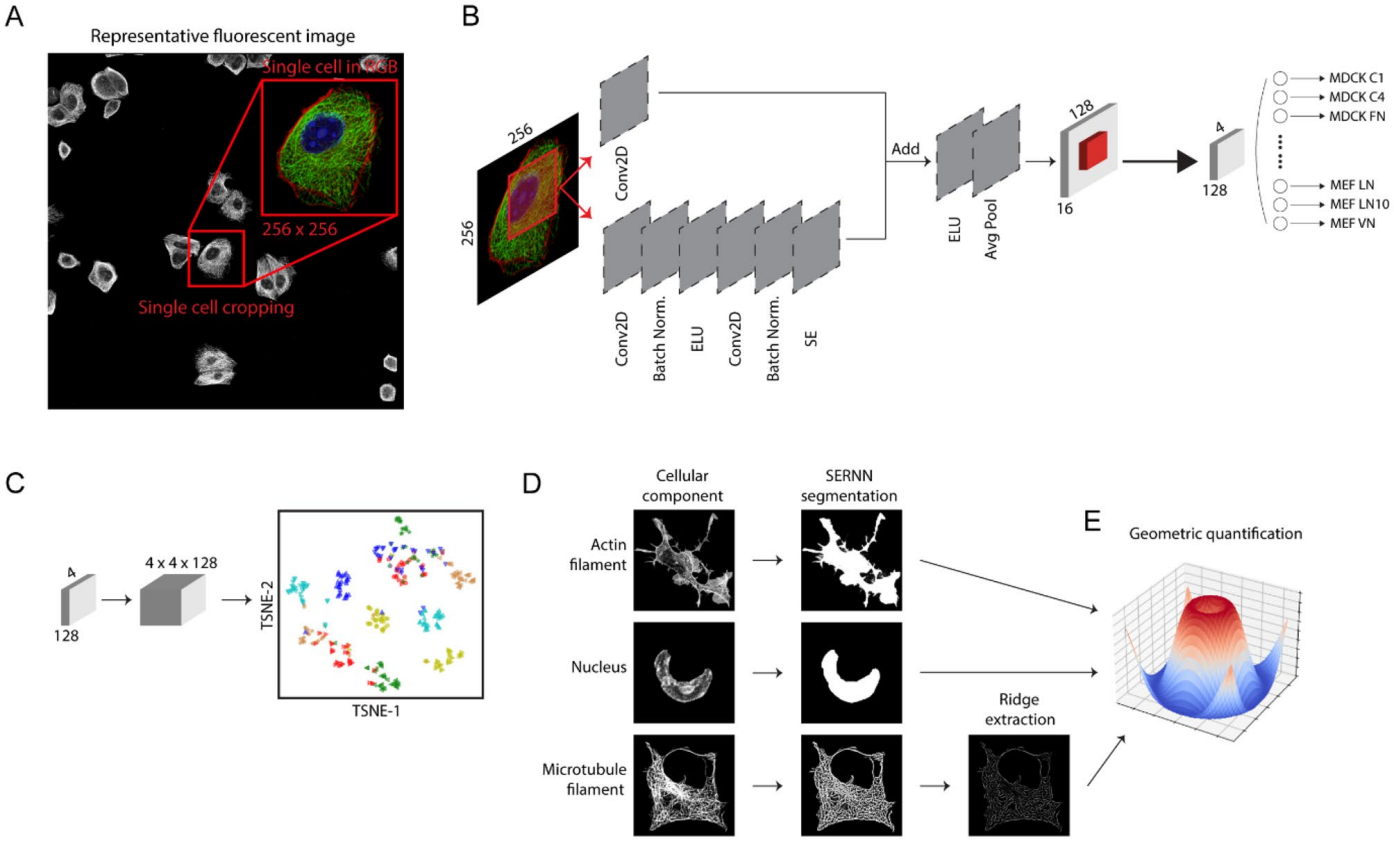
Analysis Workflow. (**A**) Images of individual cells are extracted from raw fluorescence images, processed, augmented, and then used as training data for SERNN (**B**), where the images are reduced gradually from 256 × 256 × 3 to feature maps of 4 × 4 × 128. To perform morphological profiling (**C**), feature maps are extracted, reshaped, and passed into a t-SNE algorithm to reduce into a scatter plot for cluster analysis. To perform geometric quantification (**D**,**E**), individual cellular components are segmented by a modified SERNN model. Various geometric parameters such as directionality, area, and aspect ratio are used to quantify the cellular morphology and to construct a representative morphological model for each cluster.

Although various Convolutional Neural Network (CNN) models had been particularly successful in classification accuracy^13,36,37^, from the applications of a conventional CNN model to our dataset we found that a number of architecture re-design offered significant performance advantages. We hereby developed a Residual Neural Network with squeeze-and-excitation (SE-RNN) optimized for multi-channel fluorescence image datasets, comprising an image input layer of size 256 × 256 × 3, followed by six major residual blocks, a global average pooling layer, a dropout layer, and lastly the classification layer (Fig. 2).

To train the model, a dataset containing a total of 9000 augmented images and 12 class labels were used (2 cell types × 6 ECM, Supplementary Fig. 1). The dataset was shuffled for every training phase before being split into 7:3 ratio for training and validation purposes. The model was trained a total of 5 times and the statistics from the last 10 epochs from each training phase were recorded. The average validation loss was very low at about 0.00081 ± 0.001 while the validation accuracy was very high at about 99.74 ± 0.09%. Each training phase was completed in about 320 ± 40 epochs.

**Figure 3:**
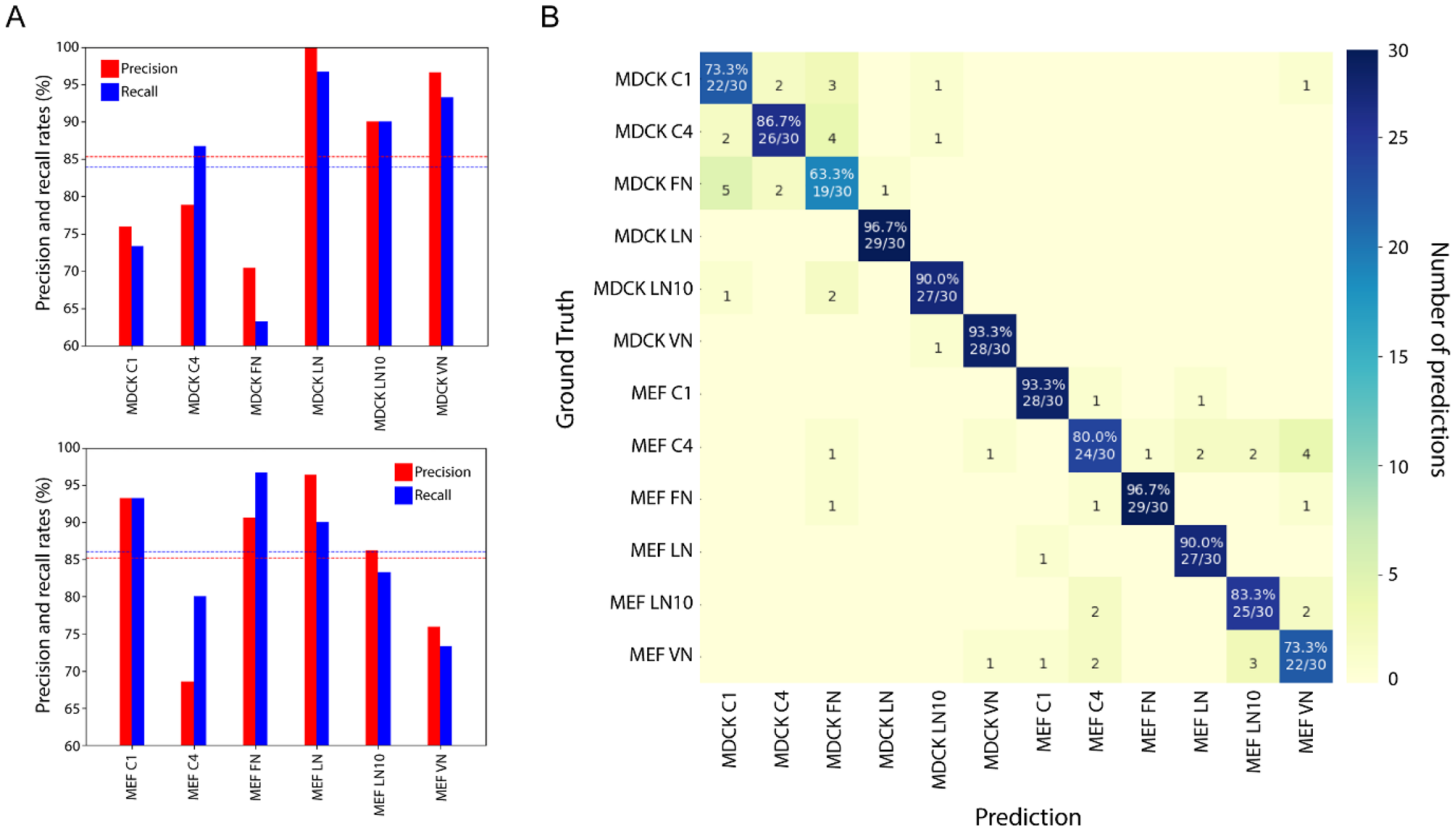
SERNN Classification performance. (**A**) Precision and recall rates for MDCK (**A**, top) and MEF (**A**, bottom) datasets. The dotted lines represent the average precision (red) and recall (blue) rates. (**B**) Confusion heatmap showing classification accuracy of SERNN model. X- and Y- axis correspond to predicted classes and ground-truth, respectively. Correct predictions (number and percentage) correspond to diagonal elements, while incorrect predictions are off-diagonal.

To evaluate the precision and recall rates of the model, the trained SERNN model was used to classify “unseen” dataset comprising 360 images. We observed an overall classification recall rate of 85% (Fig. 3). The model also achieved a high overall recall and precision rates of 85.2% and 85.0% respectively. Out of the 12 classes, the classifier achieved 90% or higher recall rate for six classes and more than 80% recall rate for nine classes, and more than 80% precision rate for more than half of the classes. Recall rates of below 80% were observed for MDCK C1 and FN and MEF VN classes. Precision rates of below 80% were also observed for these three classes and MDCK C4 and MEF C4 classes.

Next, we evaluate the performance of SE-RNN model against a conventional CNN model^13^. Both models were trained on the same dataset of 9000 augmented images. A classification test on altered images was done to evaluate the robustness of both models: 30% compression, 20% cut-out, 30% dropout, and addition of Gaussian noise with a standard deviation of 0.2 (Supplementary Fig. 2aii–v). The overall classification recall and precision rates are shown in Supplementary Fig. 2c,d. We observed that while both models performed similarly in most cases, the SERNN model showed a significantly higher precision and recall rates in tests involving Gaussian noise addition as well as 30% compression. The significant drop in classification performance involving 30% compression was expected as the details in the images were heavily distorted which significantly reduces the number of detectable features that deep learning models rely on for accurate classification. The other alterations do not result in such heavy distortions and thus the significant performance drop of the CNN model in Gaussian noise addition test was surprising. These results suggest that SE-RNN model is comparatively robust against the addition of irrelevant information and reduction of image features.

### Major roles of F-actin and microtubule channels in cell morphology classification

Next, we sought to understand the contribution of distinct subcellular component on the classification performance of our model. To do this, we reduced the intensity of a given channel by 50% and used the trained models to perform classification on the altered datasets. The dimming of a channel was to investigate the effect of the loss of information in the subcellular component on the classification results. Apart from channel dimming, other aspects of the dataset were not changed. Tests were performed using both the SERNN and CNN models where the CNN model serves as control. Supplementary Fig. 3b,c shows the precision and recall rates for both the SERNN and CNN models, revealing that both models rely heavily on the actin and microtubule channels for the identification of both cell type and the ECM. Both models exhibit more than 30% drop in precision and recall rates when either the actin or microtubule channel was dimmed, resulting in performance no better than a coin flip. This is expected given that both the actin and microtubule cytoskeletons play major roles in cell morphology. On the other hand, both models managed to achieve a respectable 70% precision and recall rates when the nucleus channel was dimmed, indicative of the minor contribution of the nucleus channel. This observation agrees with another study^21^ when they found that the position of the nucleus can be determined by the cytoskeletal fibres using a deep generative network, indicating that the cytoskeletal fibres play a much more significant role in determining the overall morphology of the cell.

### SERNN segregates cell-type specific morphological signatures and reveals ECM-specific morphological responses

**Figure 4:**
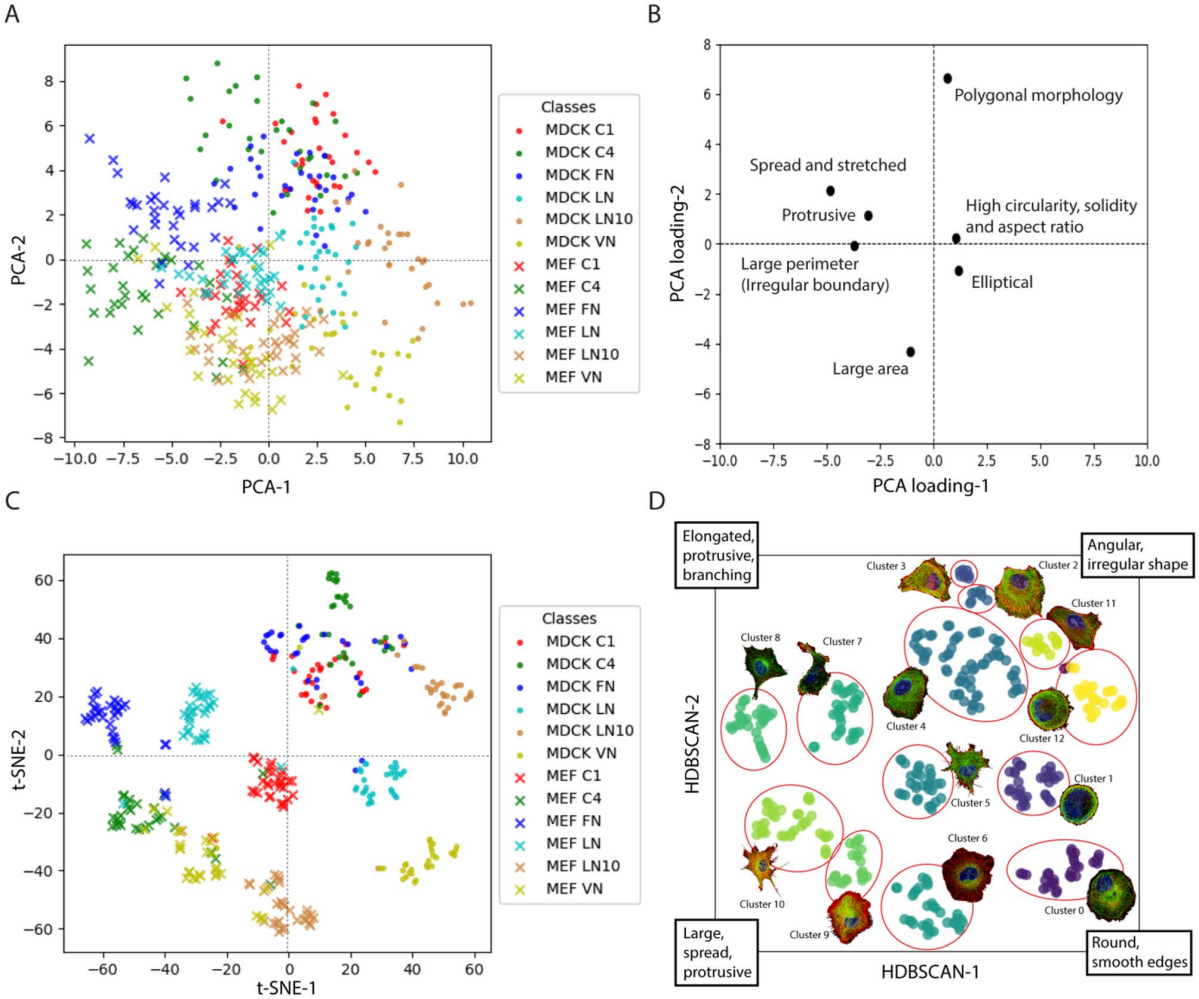
Inter- and Intra-class Morphological distinction. (**A**) Scatter plot of the first two principal components demonstrates the diverse spread of morphologies between and within cell classes. (**B**) PCA loading plot indicating how different morphological aspects of the cells influence the directions of the component values. (**C**) t-SNE dimensionality-reduced representation, emphasizing local structure. (**D**) HDBSCAN cluster analysis of t-SNE processed data from (**C**) with representative images of each cluster shown.

The performance described above suggests that the salient morphological features have been successfully learned by SERNN. To facilitate the visual evaluation of model performance, we extract the penultimate layer from the trained model and use these high-dimensional morphological feature vectors for cluster analysis as shown in Fig. 4. We first applied principal component analysis (PCA) on the extracted features to visualize the morphological diversity of our dataset (Fig. 4A). A loading plot of the PCA (Fig. 4B) shows how different morphological features in the dataset influence the direction of the component values. To investigate the quality of the extracted features, we performed cluster analysis by passing the high-dimensional features through the t-SNE algorithm for dimensionality reduction and visualization as a scatter plot (Fig. 4C). The dimensionally reduced features were subsequently clustered by HDBSCAN algorithm as shown in Fig. 4D. From the HDBSCAN plot, we identified nine homogeneous and three heterogeneous morphological categories, with representative cell images shown in Fig. 4D. As can be seen from Fig. 4A,C, epithelial MDCK and fibroblast MEF cells are clearly distinguished, indicating that our SERNN workflow can separate cell type –specific morphology.

Subsequently we examine morphological diversity within each cell types. Tree-map visualization shows that a majority of the categories were mostly homogeneous in terms of ECM condition (Supplementary Fig. 4), with a few heterogeneous clusters for MDCK and MEF comprising mainly MDCK C1, C4 and FN classes (Fig. 4D#4), and MEF C4 and VN (Fig. 4D#10), respectively. Upon close inspection of cell morphology in each of the clusters, we note that visually similar morphology is successfully grouped together. For example, rounded cells with radial “web-like” microtubule organization primarily belong to MDCK VN class (Cluster 0, Fig. 4 and Suppl. Figure 4). In contrast, cells with prominent lamellipodia belong to MEF VN (Cluster 9, Fig. 4 and Suppl. Figure 4). Thus, drastic morphological differences in how the epithelial MDCK and fibroblast MEF respond to vitronectin (VN) can be clearly distinguished by our workflow. Furthermore, extreme morphology such as highly irregular and elongated shapes with numerous actin-rich protrusions can also be clearly distinguished (MEF LN, Cluster 7, Fig. 4 and Suppl. Figure 4).

Note that we also observed a morphological class with multiple ECM conditions such as Cluster 4 (Fig. 4 and Suppl. Figure 4), which largely comprise MDCK cells from C1, C4, and FN, exhibiting angular polygonal shapes with smooth cell edges. Since the majority of MDCK C1, C4, and FN fall into this cluster, this suggests that the morphological responses of MDCK to C1, C4, and FN may be relatively similar. In contrast, for MEF, these conditions are morphologically well separated (Fig. 4C,D, MEF C1: cluster 5, MEF C4: cluster 10, MEF FN: cluster 8). MEF FN cells adopt polygonal shapes with relatively smooth edge, MEF C1 contain more actin-rich protrusions at vertices in conjunction with a relatively small polygonal shape, and MEF C4 featuring a comparatively larger area compared to MEF C1.

Taken together, our results suggest that the SE-RNN model is capable of detecting morphological similarities and differences in the dataset based on the learnt features present in the hidden layers of the model, and without relying on any predetermined set of morphological identifiers. Simultaneously, this also indicates that cell-type and ECM-specific morphological signatures are distinct and classifiable.

### SERNN-based segmentation

Next, we explore the capability of SERNN for further quantitative analysis of cellular and subcellular morphologies. By modifying SERNN model by the inclusion of a decoder network to enable image output and using adaptive thresholding to create base masks for the training samples, the modified SERNN networks can be trained to segment each cellular component as shown in Fig. 2D, where the masks are only to be used for geometric quantification. Comparison between classical segmentation methods indicates that SERNN network enable efficient segmentation of cellular components (Supplementary Fig. 5).

### Morphological interpretation of SERNN feature vectors

**Figure 5:**
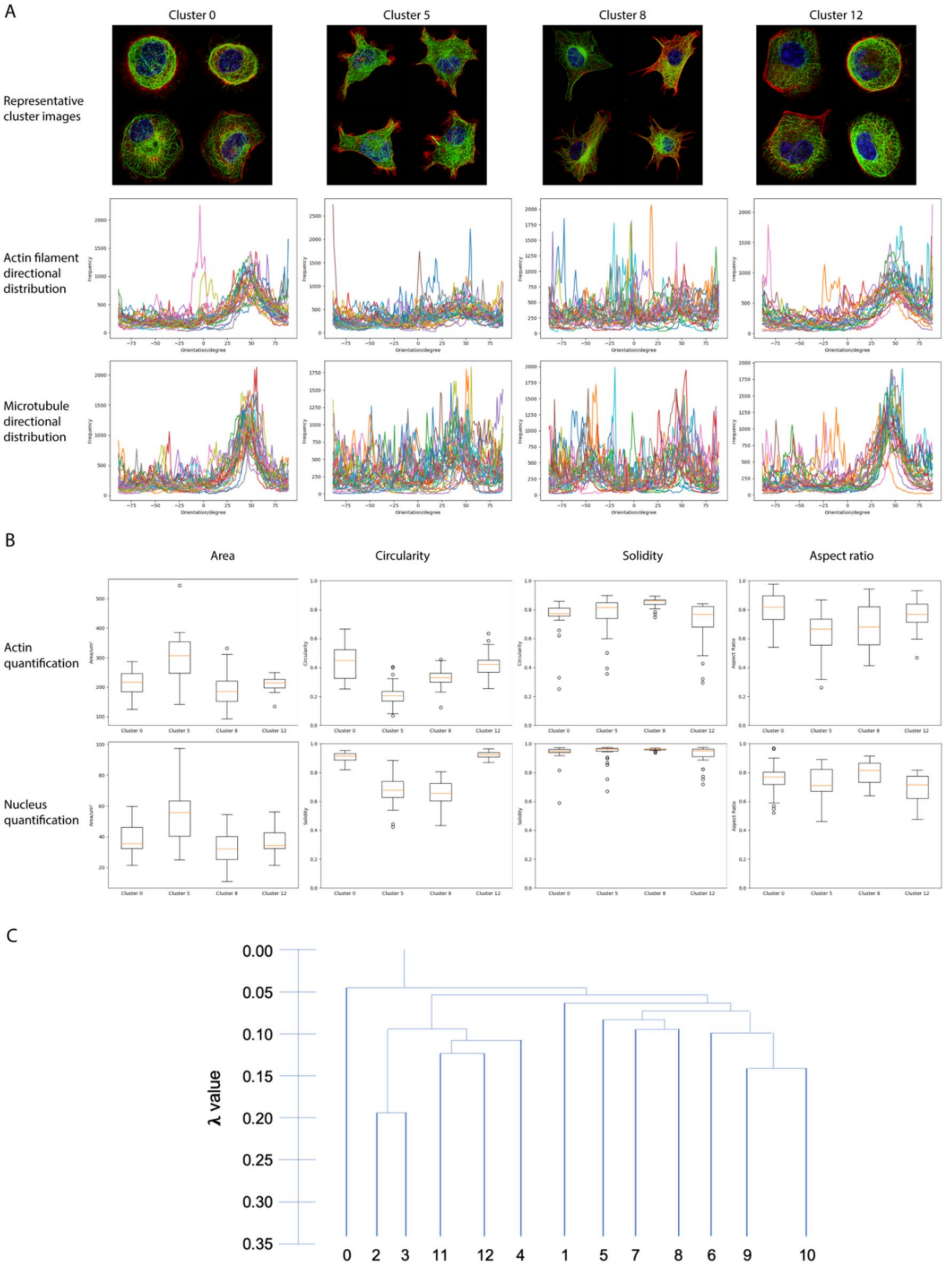
Geometric quantification of select morphological clusters. (**A**) Representative images, and directional distribution plots of actin and microtubule for clusters 0,5,8,12. (**B**) Geometric parameter quantification, showing clear differences of clusters 5 and 8 but not 0 and 12. (**C**) shows the dendrogram output using HDBSCAN’s API.

In order to gain an in-depth understanding of morphology classification process by SERNN, we focused on four clusters (#0, 5, 8, 12; Fig. 5A). These correspond respectively to MDCK (#0 and #12) with similarly rounded morphology but subtle differences in microtubule organization, and MEF (#5 and #8) with similarly irregular polygonal shapes but subtle differences in actin-based protrusions and microtubule organization. We performed a SERNN segmentation of individual cellular components (Supplementary Fig. 5). Subsequently, traditional geometric shape descriptors (area, aspect ratio, solidity, circularity) and differential geometric properties (dominant direction and orientation frequency) were calculated for both actin and microtubule filaments (Fig. 5B). As shown in Fig. 4, geometric quantitation reveals that cluster 5 (MEF) feature lower circularity and larger area, while cluster 8 (MEF) exhibited a large range in dominant direction for both actin filaments and microtubule structure. In contrast, clusters 0 and 12 (MDCK) cannot be clearly distinguished by traditional geometric and shape descriptions, likely due to additional morphological relationships that exist between the components as documented earlier^30^. Here, more complex shape descriptors such as Zernike moments may allow users to capture more complex cell morphology, but at the cost of unintuitive visualization and difficulty interpreting these phenotypes in biological terms.

**Figure 6:**
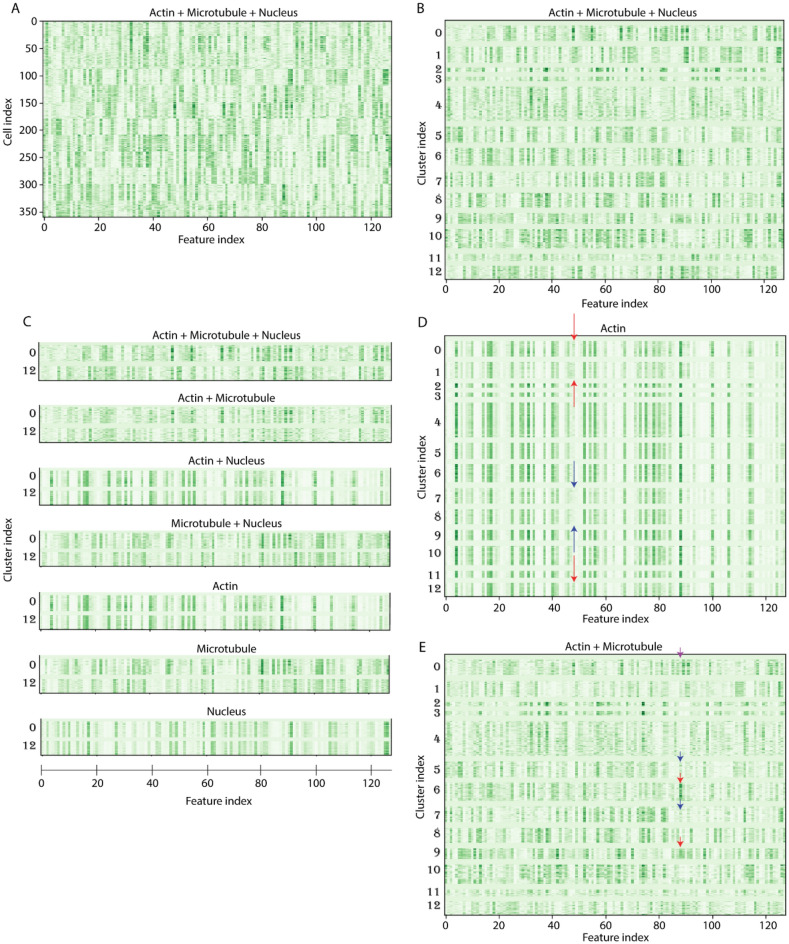
Analysis of SERNN feature vectors. (**A**) 128-dimensional morphological feature vectors from SERNN flattened layer. X-axis denotes the feature index while the y-axis denotes cell index. (**B**) Feature vectors, sorted by cluster labels (Y-axis). (**C**) Comparison of feature vectors for clusters 0 (top row) and 12 (bottom row) under channel variations are used for feature extraction, identifying microtubule channel as the main distinguishing component. (**D**,**E**) SERNN feature vectors interpretations. Cell roundness feature index is indicated by red arrows in (**D**). Roundness feature intensity is close to 0 in clusters 7 and 8 (blue arrow) which contain no rounded cells. (**E**) Mutual exclusion of actin and microtubules feature index. For clusters 6 and 9 (red arrows), mutual exclusion corresponds to extensive lamellipodia. For clusters 5 and 7 (blue arrows), mutual exclusion corresponds to filopodia-enriched actin protrusion. For cluster 0 (purple arrow), microtubules appear to be excluded from peripheral actin stress fibers. Blown-up regions of interest in (**D**) and (**E**) are shown in Supplementary Fig. 6.

To visualize the difference between clusters 0 and 12, we made use of a dendrogram of cluster hierarchy from HDBSCAN analysis (Fig. 5C). We observed that while cluster 0 branches off from the rest early on, cluster 12 bifurcates much deeper in the tree. Given that the dendrogram implies that substantial morphological differences exist between clusters 0 and 12, we next attempt to quantify these differences from the analysis of the extracted feature 128-dimensional morphological features vectors as shown in Fig. 6A,B. From the comparison of feature mappings when one or more channels were removed (Fig. 6C), we were able to determine the influence of individual and different combination of cellular components on the feature mappings. Our analysis clearly pinpointed the microtubule channel as the main distinguishing component between cluster 0 and 12, demonstrating the advantage of SERNN feature in comparison to the conventional geometric quantifications.

Encouraged by this result, we explored further whether additional morphological interpretation can be achieved using the feature maps. As shown Fig. 6D,E morphological interpretations such as roundness of the cells and mutual exclusion of actin and microtubules such as by the presence of lamellipodia can be directly interpreted from SERNN feature vectors. Altogether, we conclude that the SERNN model is capable of extracting and embedding biologically relevant morphological features and thus should be particularly useful for quantitative analysis of cell morphology under different perturbations.

### Generalization of SERNN classification to a new dataset

**Figure 7:**
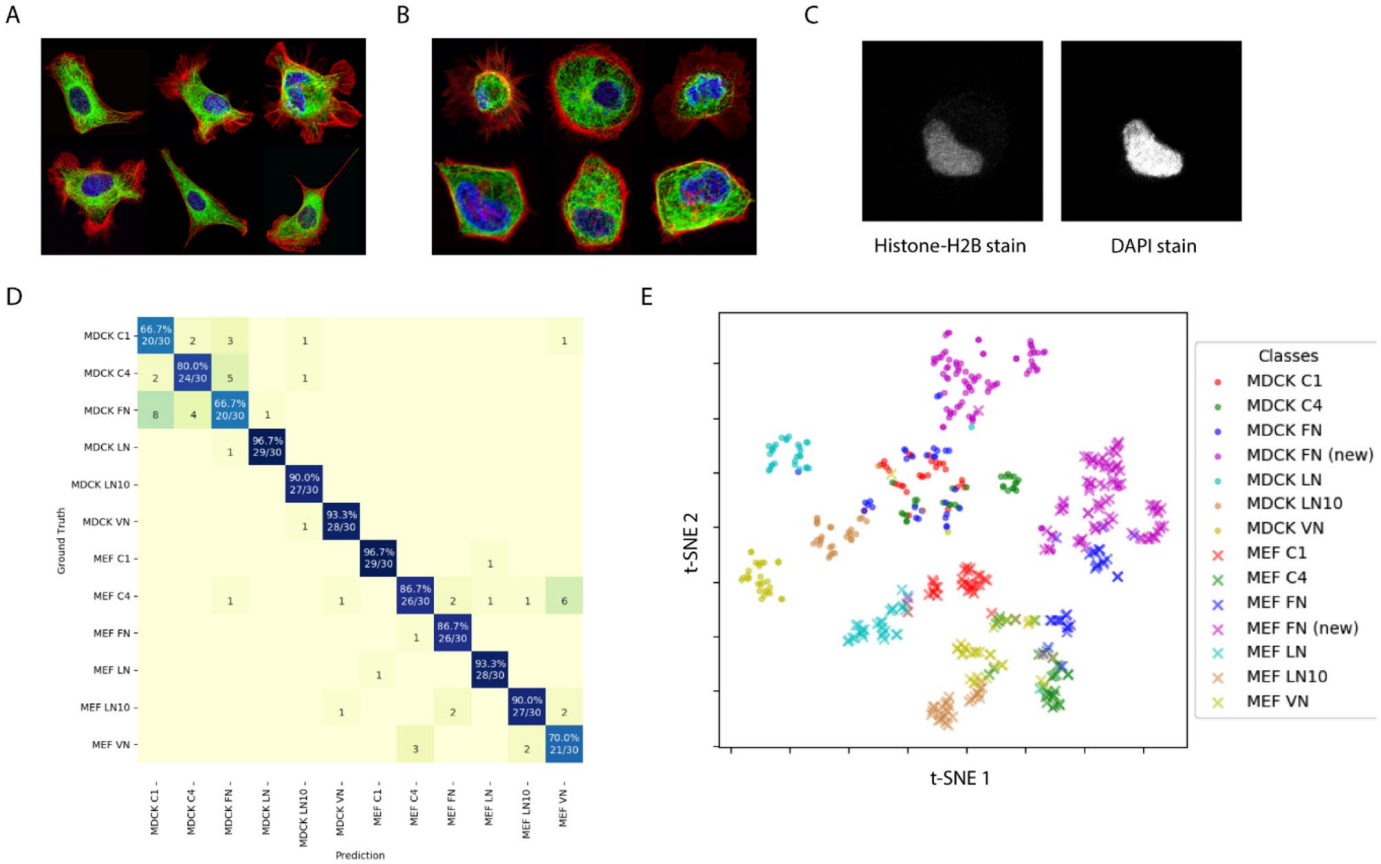
Validation of SERNN classification. (**A**,**B**) Representative images of MEF and MDCK cells, respectively. (**C**) MDCK was distinguished by stable expression of mApple-Histone-H2B, while MEF is identified by DAPI-only staining of nuclei. SERNN model is re-trained from scratch to include the new training dataset. Minimal changes in the classification results of the old test dataset are observed (**D**), indicating that the inclusion of the new training dataset does not adversely affect the model training procedure. (**E**) HDBSCAN cluster analysis of t-SNE dimensionality reduced data with additional clusters from new test data set shown in.

To investigate how our workflow can be generalized to different experimental conditions, we next applied SERNN analysis on a new dataset containing a mixture of both MDCK and MEF cells on FN. These are prepared in 3 distinct batches by different individuals, but otherwise acquired with similar imaging parameters. To establish the ground truth, MDCK with a stable expression of Histone-H2B-mApple were generated and used in conjunction with unlabelled MEF, whose nucleus is subsequently stained by DAPI (Fig. 7C). A new dataset containing 81 MDCK and 85 MEF cells were obtained, whereby 25 cells from each group are added to their respective existing FN classes for training, while the remainder were used as the new test dataset. The revised trained model was then applied on the original test dataset. As shown in Fig. 7D, the overall classification accuracy remained high, indicating that the inclusion of the new training data did not appreciably affect model training and that the model was robust to new training samples. We also tested the newly trained model on the new test dataset, observing a very high classification accuracy of 98%. Overall, the high classification accuracy on both the original and new test datasets corroborated the ability of trained SERNN model to distinguish between different cell types on different ECM. As above, we extracted the feature vectors from the original and new test datasets and performed cluster analysis on t-SNE reduced vectors (Fig. 7E), with a total of 24 clusters obtained.

While our validation experiments show that the SERNN feature vectors can be used as a basis for morphological cluster analysis for differential morphological quantification, our analysis also identify a number of limitations. This generally stems from the choice of clustering algorithm used. As shown in Supplementary Fig. 7A, HDBSCAN was able to produce largely sensible clusters with the exception of cluster 15, which had a higher degree of intra-cluster morphological heterogeneity as compared to the other clusters. It had been earlier noted that the default cluster selection method, known as “eom” (Excess of Mass), has a tendency to yield one or two large clusters and numerous small clusters^38^, in contrast to the alternate “leaf” cluster selection method which selects the leaf nodes from the cluster tree hierarchy and produce more fine-grained clustering. Indeed, “leaf” HDBSCAN clustering led to much more sensible clusters such as clusters 22 and 35 in Supplementary Fig. 7B, which were initially clustered together (cluster 15 in Supplementary Fig. 7A). Such issue is likely due to scalability issue, where the clustering results tend to be less sensible with increasing data density since clustering algorithm relies on a distance metric to determine the cluster boundaries. An alternative is to increase the dimensionality of the dataset to be clustered—instead of using only two dimensions, three or more dimensions could be used. Increasing the dimensionality of the dataset may lead to more separation between each instance and thus allow for more sensible clustering results. However, with increasing dimensionality, it becomes challenging to perform a visualization plot for analysis and consequently difficult to determine a suitable clustering algorithm to implement. Furthermore, it is also difficult to determine the exact number of dimensions where the instances are spread out far enough from one another but not too far. It is well recognized that clustering algorithms, being classical machine learning techniques, do not work well with high dimensionality data due to a problem known as the curse of dimensionality^39^. Other parameters such as the min_cluster_size can also be adjusted to avoid large clusters, but the search for the optimal values for these parameters will require a trial-and-error process and is beyond the current scope of this study.

Lastly, we demonstrate the quantification of morphological differences (Supplementary Fig. 7C) using two clusters comprising the new datasets (clusters 36 and 39) and another cluster comprising the old dataset (cluster 22). Visual analysis shows that clusters 22 and 36 are morphologically similar with the main difference being the microtubule density. Cluster 39 is significantly different as prominent lamellipodia is observed rimming the rounded cells. Comparison of the feature vectors between clusters 22 and 36 show that they are largely similar, with the red box highlighting the features that likely correspond to the differences in microtubule intensity. Comparison of the feature vectors between cluster 39 and the other two clusters show significant difference, as highlighted by the blue boxes. However, from this comparison alone, we were unable to determine which features specifically refer to the presence of lamellipodia and/or roundness. Further comparisons to the feature vectors of other clusters can be performed to interpret the differences, but this can be time-intensive and also prone to bias error. A literature survey shows that there is a lack of research in this area, and thus further work is needed to develop methods that can automate the interpretation process. A technique to reconstruct the image using the extracted features will also be useful in highlighting the morphological aspects of the cells, which will further improve the interpretability of the feature vectors.

## Discussion

In this study we developed a data-driven approach to aid in the quantification of morphological complexity of animal cells. We focused on multi-channel fluorescence microscopy as it is a robust, well-established, and broadly accessible imaging modality and apply our approach to address the correlation between ECM and cell shape, which can be broadly perceived by visual inspection at qualitative level but has been challenging to computationally analyze in a generalizable manner. We described SERNN, a deep learning-based computational architecture, which is capable of automated morphological classification of multi-channel cellular images with high performance and reliability. Our analysis showed that that the wide variety of morphology on different ECM exhibited by different cell types can be robustly classified by cluster analysis on high-dimensional morphological feature vectors extracted from the SERNN model. Our validation experiments demonstrated that our model was capable of generalizing to different datasets. Moreover, in addition to enabling meaningful cluster analysis, SERNN can also be utilized in differential morphological quantification and segmentation.

Limitations of our analysis include the evaluation of the clusters, which can be time-consuming and prone to bias error. While there exists numerous metrics to evaluate quality of clusters, they are only suitable for low-dimensional data. At present, a metric that can properly capture the similarity or dissimilarity between high-dimensional data in a cluster has been lacking. Cluster 15 in Supplementary Fig. 7A highlights this issue—the cluster was determined by various metric to have a high degree of similarity, but visual analysis showed that there was significant intra-cluster heterogeneity. In fact, it is not surprising that the metric will return such a positive result as this was expected when analysing the visualization plot in Fig. 7E, where the instances lie very close to one another. Therefore, there is a need to develop a metric to effectively quantify the differences between high-dimensional instances. Alternatively, it is possible to use Fréchet inception distance (FID) to evaluate the clusters provided the clusters themselves contain a large number of images, typically in the range of thousands. Another limitation of our analysis includes the scalability of the clustering algorithm, where the algorithm does not perform as well with high data density. Increasing the dimensionality of the dataset may help to separate the instances, but it becomes challenging to visualize them and consequently difficult to pick a suitable clustering algorithm. This is an inherent challenge with a large number of features and clusters. Lastly, there is a need to improve the interpretability of the feature vectors. One possible approach would be to reconstruct the cell image using the extracted feature vectors to highlight the morphological aspects of the cells. An alternative is to implement Grad-CAM^40^ for visual analysis but expand it to be able to work on individual channels for multi-channel images.

In our present analysis, we chose to use the actin filament, microtubules, and the nucleus from fluorescence confocal microscopy as test cases. The actin cytoskeleton confers structural rigidity and predominantly contribute to cell shape regulation^41,42^. The microtubules are important for cell polarity and signalling networks governing cell migration, while their network organization are in turn dependent on cell signalling states^43,44^. The nuclear morphology and positioning are significantly influenced by the cytoskeleton where the perturbation of the actomyosin and microtubules will exert tensile and compressive forces on the nucleus, changing its size and shape. However, there is no a priori limitation against future incorporation of other organelles. Additional cellular structures such as the Golgi apparatus or focal adhesions could be incorporated for more extensive morphological profiling. With increasing number of channels being used for profiling, the training time will increase, and computational hardware becomes the limit as the amount of memory required to run the model increases. As such, while there is no theoretical limit on the number of cellular structures to be used for an extensive morphological profiling, it is equally important to consider the structures required for the study and the computational resources available for model training. Dynamic analysis is particularly useful in studying the morphodynamics changes and is an area of future investigation. Long-term fluorescence imaging is, however, inherently affected by photostability which limit the observation duration. To overcome this, amalgamation of images from different modality such as phase contrast or quantitative phase imaging^45^, preferably on the same microscope platform, may be particularly useful^46^. Additionally, it may be instructive to compare or integrate our approach with recent studies making use of deep learning to perform artificial fluorescent labelling^47^.

Biologically, our results underscore that cell shapes are apparently non-random and can be classified into robust morphological categories in a cell-type and ECM-specific manner. Interestingly, each experimental condition is marked by a certain level of morphological heterogeneity in which several morphological profiles can be observed. What give rise to such heterogeneity remains to be further investigated. These may include intrinsic genetic heterogeneity in cell culture population, which could be addressed by subjecting these cells to clonal selection. Alternatively, since progression through the cell cycle is known to influence general cell morphology as well as cell adhesion and cytoskeleton^48^, cell cycle synchronization or the use of cell cycle reporter^49^ may help elucidate the underlying correlation further. All in all, given that the number of morphological clusters observed are in the range of several, it is hopeful that the underlying biochemical signalling differences that underlie such heterogeneity will be tractable. In parallel to dissecting the origin of morphological heterogeneity, further experimentation could also explore the contribution of ECM density, substrate rigidity or viscoelastic properties, as well as spatial organization both at the micro- and nano- scale, in contributing to the morphological profiles of the cells^50,51^.

Altogether, we envision that our analysis workflow will be a valuable tool for dissecting cellular morphodynamics. For example, our analysis can be harnessed in combination with genetic and biochemical perturbations to help develop experimentally testable hypothesis that elucidate the linkage between cell morphology and cellular signalling states. Our approach is currently designed for in vitro cell culture studies, which provide controllable conditions that allow for well calibrated analysis. However, further development may enable the extension of this approach to probe tissues in vivo. In particular, ECM in vivo is typically a complex mixture of variable composition, abundance, modification, and mechanical conditioning^52^. Given that cells have the inherent ability of cells to sense the underlying ECM, if the relationship between cell shape response and ECM microenvironment can be established in well-calibrated experiments, we envision that imaging-based analysis of cell shape in vivo could be useful as a direct read-out of ECM properties with advantages in cost, accessibility, and versatility.

## Materials and methods

### Cell culture and specimen preparation

MEF (generous gift from Michael P. Sheetz, Mechanobiology Institute, Singapore) and MDCK (generous gift from Benoit Ladoux, University of Paris, Diderot, France) cells were cultured in a 5% CO_2_, 37 °C humidified atmosphere in high-glucose DMEM-GlutaMAX medium (Life Technologies) supplemented with 10% fetal bovine serum (FBS, Life Technologies), 1% sodium pyruvate and 1% penicillin/streptomycin (P/S, Life Technologies). Glass coverslips were cleaned and sterilized by ethanol and UV (25 min) before coating. Cleaned coverslips were coated with 10 μg/mL bovine fibronectin (F1141, Sigma); 15 μg/ml Vitronectin (08–126, Sigma); 30 μg/ml Collagen I (5005, Advanced Biomatrix); 30 μg/ml Collagen IV (354,233, BD Bioscience); 10 μg/ml Laminin (23,017–015, Invitrogen); 10 μg/ml Laminin-10 (T303, Takara) for 1 h at 37 °C and washed 3 times with 1X PBS. For immunofluorescence staining, cells were plated on different ECM-coated coverslips in serum-free media. After plating for 2 h, cells were washed with 1X PBS and fixed with PFA 4% for 10 min. Then, cells were permeabilized with 0.1% Triton X-100 for 3 min. After washing with PBS, cells were incubated with 5% BSA for 1 h for blocking, Fixation, permeabilization and blocking were performed at room temperature. Subsequently cells were incubated with primary antibody for α-tubulin (ab7291, Abcam) at 4 °C, overnight. After washing, cells were incubated with secondary antibody of Alexa Fluor 488-conjugated donkey anti-mouse IgG (A21202, Life Technologies), DAPI (D3571, Life Technologies) and Alexa Fluor 568-conjugated phalloidin (A12380, Life Technologies) for 1 h at RT. After final washing, cells were mounted in DAKO fluorescence mounting medium (s3023, Agilent).

### Confocal microscopy

Laser-scanning confocal fluorescence microscopy images was performed using Nikon A1R laser-scanning confocal microscope equipped with 100 X 1.4 NA objective lens and running Nikon NIS-Elements acquisition software. Laser excitation wavelengths used are 405 nm, 488 nm, and 647 nm for nucleus, microtubule, and F-actin, respectively. Images were captured with identical settings except for different zoom ratio for MDCK and MEF, yielding a pixel size of 80 and 120 nm, respectively. As we aim to study the differences in morphological properties between MDCK and MEF cells, we prioritize the clarity of the cellular components by changing the zoom ratio over using the representative sizes of the cells themselves which can result in images of very small cells with large amounts of blank spaces that do not contain any useful data. By reducing the amount of sparsity in the dataset, this will also ensure a more meaningful model training. To control against variations in specimen preparation, only samples with uniformly consistent staining were used for imaging and subsequent analysis.

### Image processing

From large-area confocal microscopy images, regions containing individual cells were manually cropped, centred, and padded to 256 × 256 pixels dimension. Cropped images were denoised by Gaussian filtering with a kernel size of 3 × 3 and a standard deviation of 1.0. Image contrast was adjusted by histogram equalization. Subsequently, the processed images were augmented via geometric and arithmetic means to simulate different cell orientations and sub-optimal imaging conditions, respectively. Geometric augmentations used include image rotation, mirror, and scaling while arithmetic augmentations used include additive Gaussian noise and image dropout. Augmented images were converted into RGB (R: F-actin, G: microtubule, B: nucleus). The intensity of each channel was then normalized to a [0, 1] range using the formula $\widehat{I}=\frac{I-\mathrm{min}\{I\}}{\mathrm{max}\left\{I\right\}-\mathrm{min}\{I\}}$, where I refers to the raw intensity of the image and Î refers to the normalized intensity. Images were labelled according to the cell type and the ECM substrate with a total of 12 classes (2 cell types × 6 ECM), with each class containing 750 training images for a total of 9000 training images. Note that only the augmented images were used for training the model and that only the real images were used in the cluster analysis.

### Statistical analysis of cell morphological differences

Visual analysis was first performed directly on the non-augmented dataset, with findings indicating that there were distinct morphological differences. We proceeded to perform a F-test to investigate if these findings were statistically significant. PCA was implemented directly on the RGB single cell images (including all 3 cellular components) to extract the first two principal components. Using these two components, we performed an F-test to determine whether there were any significant statistical differences between the different classes. The test returns two results: (1) ratio of the variation between sample means to variation within the samples, (2) p-value which is used to reject or accept the null hypothesis that indicates that the sample means are the same. The null hypothesis is rejected if the p-value is lower than the significant value of 0.05. The full results are shown in supplementary table 1. The results of the F-test indicated that there were significant statistical differences, and thus we carried out experiments to further investigate these differences.

### Residual neural network with squeeze-and-excitation (SERNN)

SERNN network comprises an image input layer of size 256 × 256 × 3, followed by six major residual blocks, a global average pooling layer, a dropout layer, and lastly the classification layer. Stochastic gradient descent was used as the learning algorithm and the initial learning rate was set to 0.001. A learning scheduler was implemented to reduce the learning rate by a factor of 0.9 if the validation loss parameter does not improve by 0.001 after 25 epochs. An early stopping algorithm was implemented to stop the training and restore the best weights if the validation loss parameter does not improve by 0.0001 after 50 epochs. 70% of the augmented dataset was used as training set while the remaining 30% was used as validation data. A batch size of 16 was selected for the training. The training was performed using the GPU (Nvidia GeForce RTX 2080 Ti 11 GB) on a Windows workstation with Intel® Xeon® W-2123 CPU @ 3.60 GHz processor and 64 GB of RAM.

### Extraction of morphological features

Morphological features of the dataset were extracted from the last third layer of SERNN model wherein the morphology of each cell is described by a 128-dimensional vector. Dimensionality reduction of the morphological feature vectors was performed using t-SNE algorithm. The parameters for t-SNE were adjusted from initial parameter set as in^53^, resulting in the following parameters, n_components: 2, perplexity: 2, early exaggeration: 4, random_state: 42, learning_rate: total number of cells / 12. The reduced vectors were then clustered using the HDBSCAN algorithm with the default parameters of minimum cluster of size of 5.

### SE-RNN-based automated segmentation of cellular structures

SERNN model was modified to perform the automated segmentation of cellular structures via the addition of a decoder network to the existing encoding network. The encoding network transforms the input image into a high dimensional feature vector, which is fed into the decoding network to be transformed back into an image. In our case when the model is trained, the encoding network transforms the processed micrograph into a feature vector, and the decoding network transforms the vector into an output probability map where the intensity of each pixel is between 0 and 1.

To train the modified SERNN model, images of cellular component and the target segmented binary mask are used as paired training data. Segmented binary masks were obtained by adaptive thresholding of cellular component images, followed by manual processing in ImageJ to improve segmentation quality. 50 training sets were produced for each cellular component, and data augmentation was used to produce a total of 1350 training sets. The training sets were split into a 7:3 ratio for training and validation purposes respectively. Training parameters were similar to those used in the initial SERNN training. The output probability maps were thresholded to remove pixels with intensity values below 0.2. The thresholded maps for the actin filaments and nucleus are further processed to remove small and detached components and flood-filled to remove missing pixels within the segmented components. For microtubule channel, Frangi ridge operator^54^ was implemented onto the thresholded maps to extract the ridge-like microtubule filaments.

### Geometric quantification of cellular components

Various geometric quantifications of the cellular components were performed to study the morphology of the components. This includes the quantification of the physical space—area and perimeter, ratio—aspect ratio, solidity and circularity, and differential geometry which investigates the dominant orientation and orientation frequency of the cellular component. The first two types of quantification were done using the “Analyze Particles” function in ImageJ implemented on binary images, while the latter was done using the OrientationJ plugin for ImageJ implemented on raw micrographs^55^.

### Metrics and descriptors used in the study

#### Evaluating classification performance

Using an example where a dataset contains two classes and analysing the results that have been classified as class 1:

$$ {\text{True positive }}\left( {{\text{TP}}} \right) \, = {\text{ Instance correctly classified as class 1 when ground truth is class 1}} $$

$$ {\text{False positive }}\left( {{\text{FP}}} \right) \, = {\text{ Instance incorrectly classified as class 1 when ground truth is class 2}} $$

$$ {\text{False negative }}\left( {{\text{FN}}} \right) \, = {\text{ Instance incorrectly classified as class 2 when ground truth is class 1}} $$

These three measures can be used to form the following metrics for evaluating classification performance:

Precision = $\frac{TP}{TP+FP}$, which refers to the number of correct classifications over the total number of predictions for the class. This measures the relevance of the classification results. For example, given a class of 30 MDCK C1 cells, 20 of them were classified correctly and 5 other cells were incorrectly classified as MDCK C1—this translates into a precision rate of (20)/(20 + 5) = 20/25 = 80%.

Recall = $\frac{TP}{TP+FN}$, which refers to the number of correct predictions for the class. This measures the ability of the model to determine the relevance of an instance. For example, given a class of 30 MDCK C1 cells, 20 of them were classified correctly while the other 10 were classified wrongly as other classes—this translates into a recall rate of (20)/(20 + 10) = 20/30 = 66.7%.

These two metrics have been chosen as they are useful in evaluating the model’s classification performance and are also easily interpretable by users.

#### Geometric quantification

We quantified the morphology of the individual cellular components using selected traditional cell shape descriptors:

1. Area and perimeter were used to quantify the physical space that the components occupy
2. Aspect ratio, solidity, and circularity were used to describe the overall shape
3. Differential geometry was used to describe the dominant direction and orientation frequency of the actin and microtubule filaments.

## Supplementary Information

Supplementary Information.

## Data Availability

Plots were generated using Python’s libraries—Seaborn 0.11.1 and Matplotlib 3.1.3. All figures were generated using Adobe Illustrator 2022. The data and material generated during and/or analysed during the current study are available from the corresponding author on reasonable request.

## Code availability

The analysis code can be found in the following link: https://github.com/KanchanawongLab/Unsupervised-Morphological-Quantification. Instruction manuals regarding the installation of Python and dependencies as well as a guide to using the software are also provided in the same repository.
