# Supplementary material for: Self-supervised classification of subcellular morphometric phenotypes reveals extracellular matrix-specific morphological responses
Source: Sci Rep. 2022 Sep 12;12:15329. doi: 10.1038/s41598-022-19472-2 (PMC9468179; doi:10.1038/s41598-022-19472-2)
Supplement: Supplementary file 1 — Supplementary Information. [file 41598_2022_19472_MOESM1_ESM.pdf]

Supplementary Materials

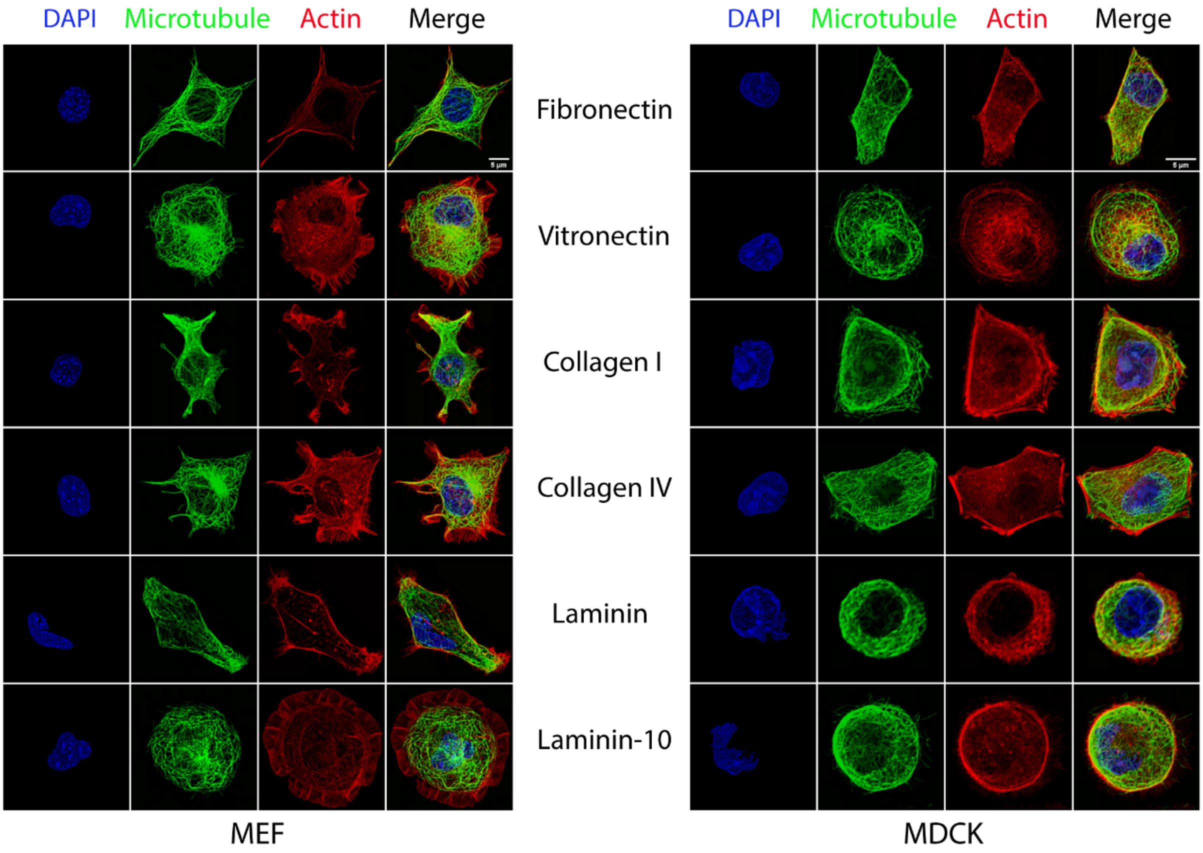

Supplementary Figure 1: Immunofluorescence of MEF and MDCK cells on various ECM. 12 classes (2 cell types, 6 ECM proteins) used in this study are shown with the representative cell image in merged RGB alongside its individual channels – nucleus, microtubule filament, and actin filament. Scale bar: 5 μm

**Statistical Analysis (F-test)**		
ECM	*F-score*	*P-value*
Collagen I	182.7	3.69×10^{-28}
Collagen IV	32.54	5.61×10^{-8}
Fibronectin	184.4	2.46×10^{-28}
Laminin	501.0	7.22×10^{-51}
Laminin-10	14.15	2.37×10^{-4}
Vitronectin	47.76	1.09×10^{-11}

Supplementary Table 1: F-score and p-value from PCA analysis of MDCK and MEF datasets for every ECM condition. The null hypothesis is rejected if the F-score is above the F critical value and the p-value is smaller than 0.05.

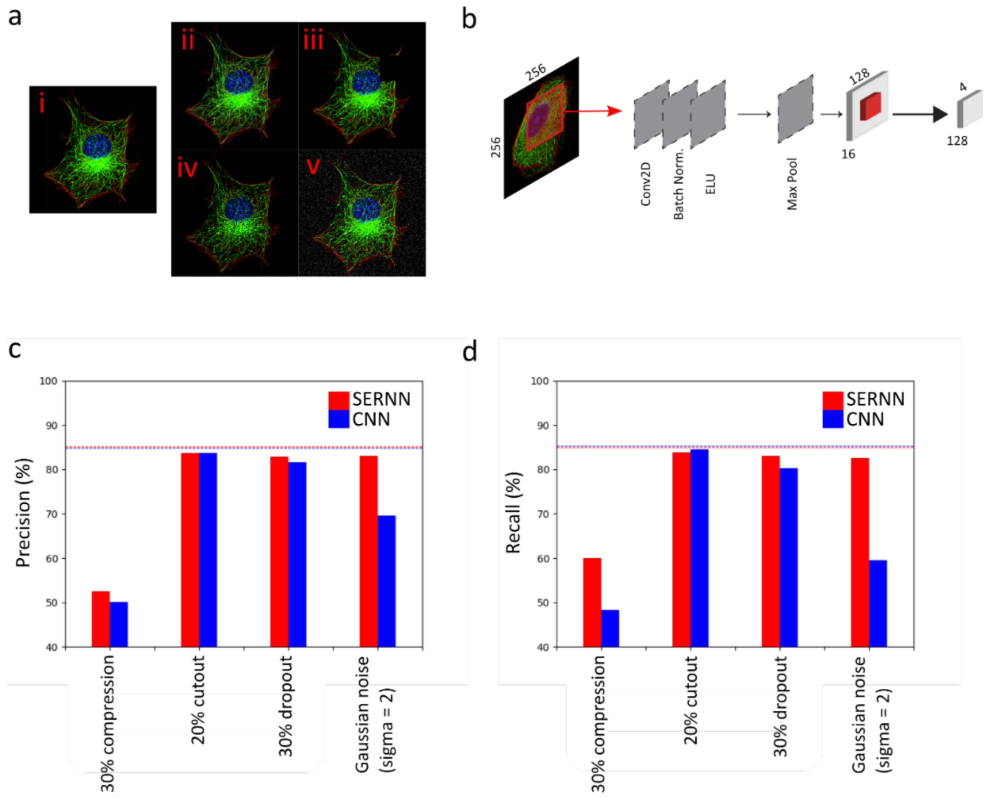

Supplementary Figure 2: (a) shows the alterations done on the test images: (ai) original image, (aii) 30% compression, (aiii) 20% cutout, (aiv) 30% dropout, (av) Gaussian noise with a standard deviation of 2. (b) shows the architecture of the CNN model used in the comparison. (c) and (d) show the precision and recall rates, respectively, of both the SERNN and CNN models on the altered test images. The dotted lines indicate the precision and recall rates on the unaltered images.

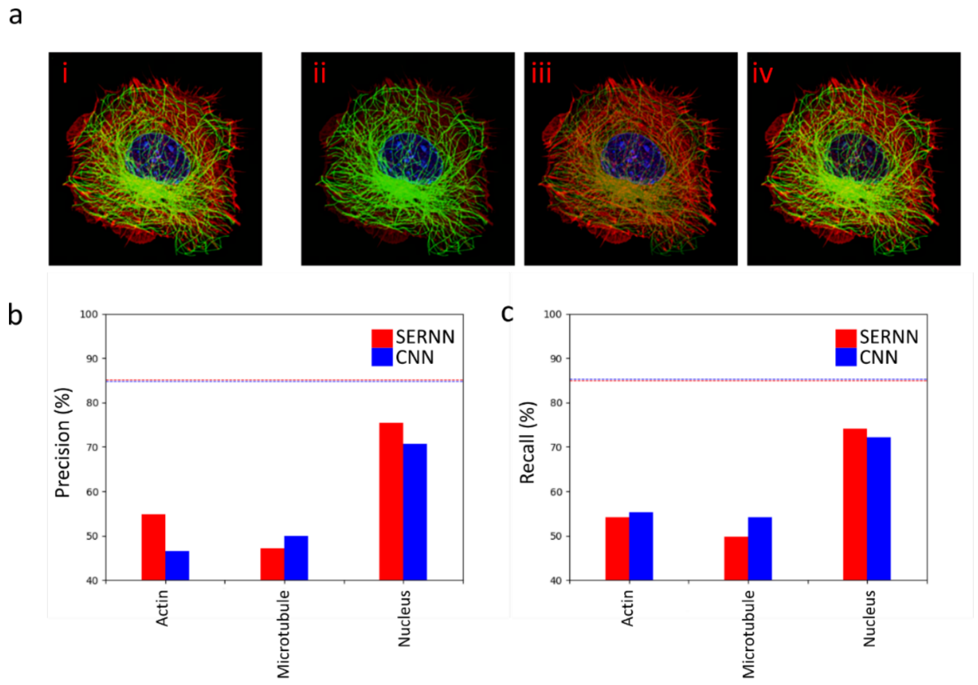

Supplementary Figure 3: (a) shows the alterations done on the test images: (ai) original image, dimming of the (aii) actin, (aiii) microtubule, (aiv) nucleus. The dimming of the channel is done by reducing the intensity by 50%. (b) and (c) show the precision and recall rates, respectively, of both the SERNN and CNN models on the altered test images. The dotted lines indicate the precision and recall rates on the unaltered images.

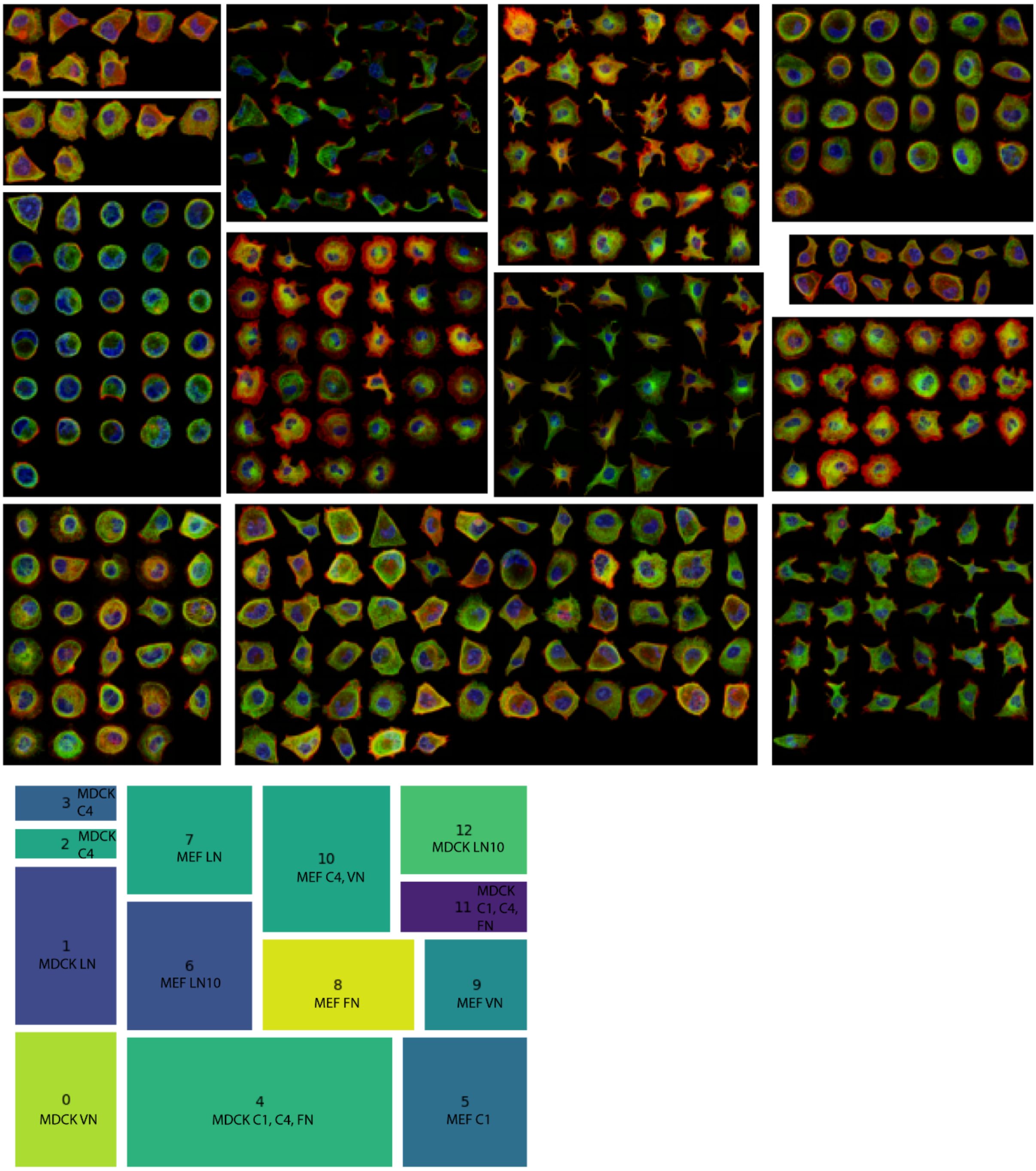

Supplementary Figure 4: Treemap representation of the HDBSCAN cluster results (top) and the respective labels for each cluster (bottom). The labels also include the majority cell type and ECM conditions found in each of the clusters.

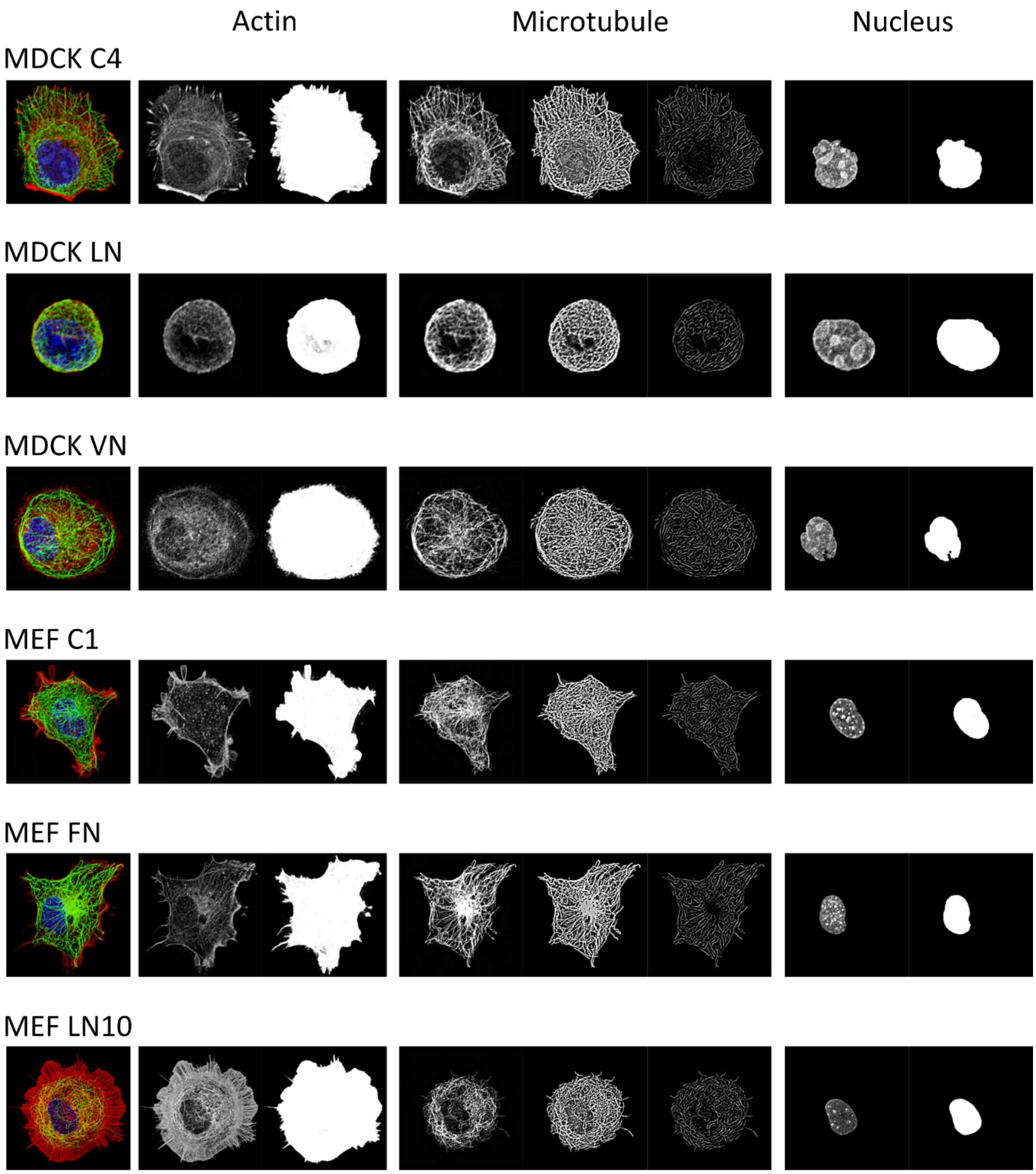

Supplementary Figure 5: SE-RNN Segmentation for different conditions. Column definition: (from left to right) original image in RGB, actin, segmented actin, microtubule, segmented microtubule, extracted microtubule using Frangi operator, nucleus, segmented nucleus. All the segmented results shown here are unprocessed.

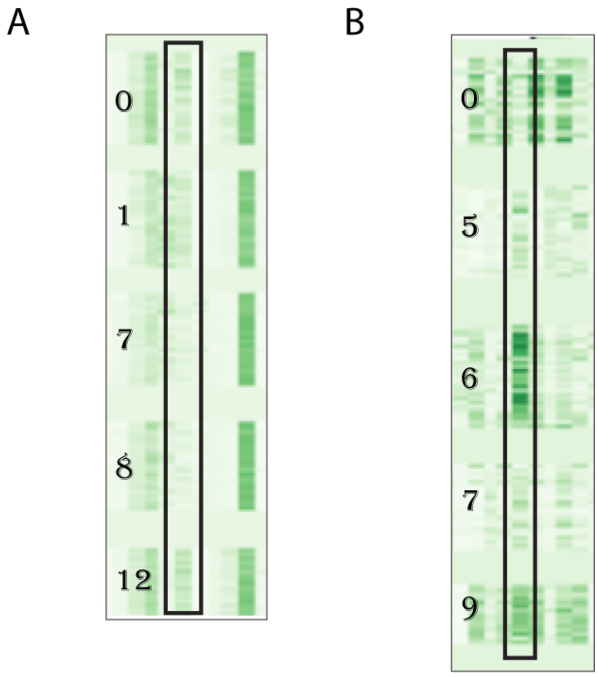

Supplementary Figure 6: SERNN Feature Interpretation.

(A) and (B) Zoomed-in of regions of interests from figures 6D and 6E respectively. The important feature index is highlighted by a black box.

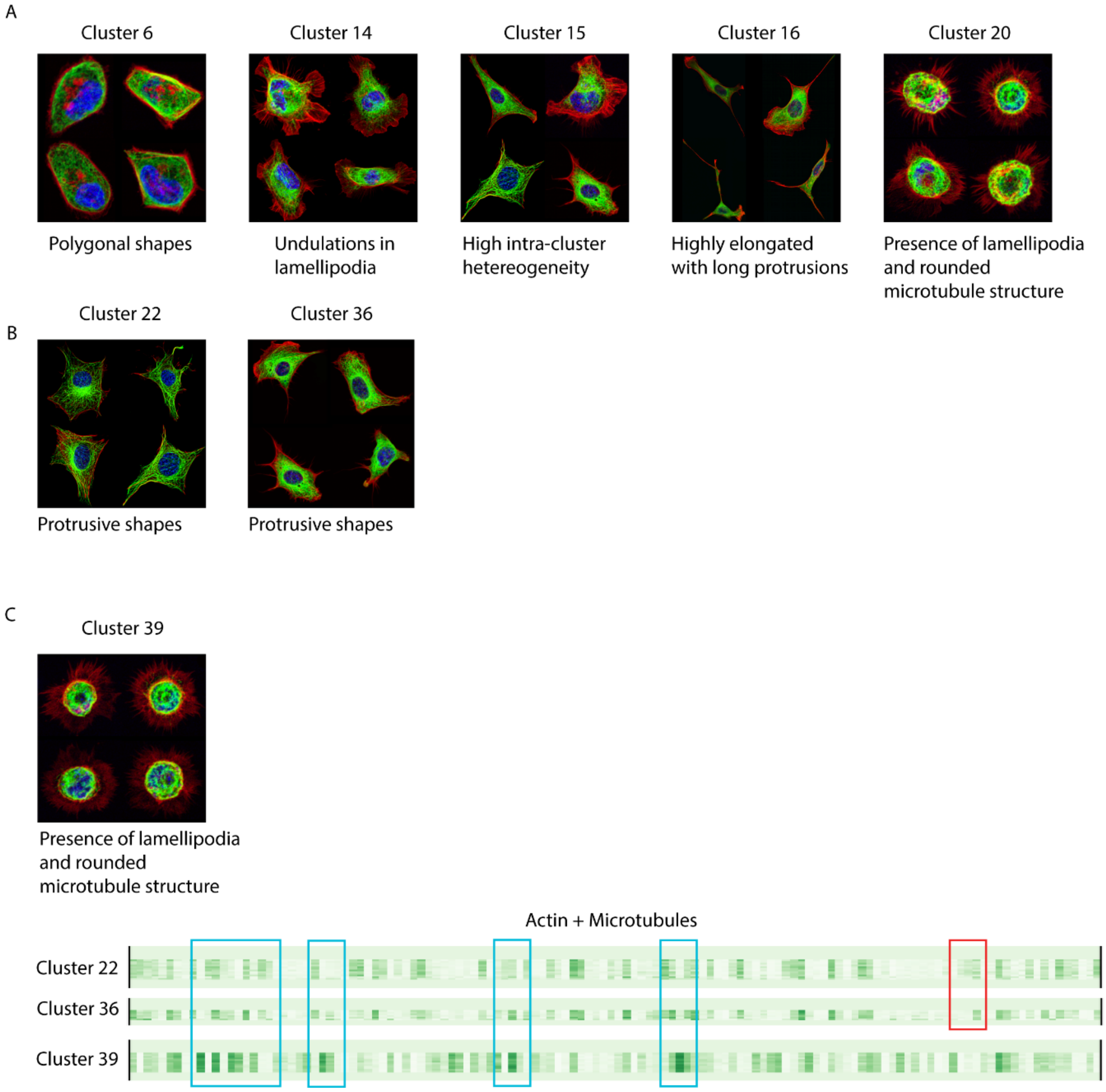

Supplementary Figure 7: In-depth interpretation of SERNN classification

(a) Initial clustering results, where cluster 15 has high intra-cluster heterogeneity. (b) Examples of more fine-grained clusters from cluster 15 after changing one of HDBSCAN's parameters. (c) Feature vectors of clusters 22, 36, and 39 being used for quantification of morphological differences.
